# Stable isotopes are quantitative indicators of trophic niche

**DOI:** 10.1111/ele.13374

**Published:** 2019-08-28

**Authors:** Harry H. Marshall, Richard Inger, Andrew L. Jackson, Robbie A. McDonald, Faye J. Thompson, Michael A. Cant

**Affiliations:** ^1^ Centre for Research in Ecology, Evolution and Behaviour, Department of Life Sciences University of Roehampton London UK; ^2^ Environment and Sustainability Institute University of Exeter Penryn UK; ^3^ Department of Zoology, School of Natural Sciences Trinity College Dublin Dublin Ireland; ^4^ Centre for Ecology and Conservation University of Exeter Penryn UK

**Keywords:** Banded mongoose, diet, isotopic niche, niche overlap, stable isotopes, tissue integration time, trophic niche

## Abstract

Hette‐Tronquart (2019, Ecol. Lett.) raises three concerns about our interpretation of stable isotope data in Sheppard *et al*. (2018, Ecol. Lett., 21, 665). We feel that these concerns are based on comparisons that are unreasonable or ignore the ecological context from which the data were collected. Stable isotope ratios provide a quantitative indication of, rather than being exactly equivalent to, trophic niche.

## Introduction

Hette‐Tronquart (2019) raises three concerns about our study demonstrating that increased intragroup competition predicts higher individual foraging specialisation in banded mongooses *Mungos mungo* (Sheppard *et al.* 2018). We address each of these concerns in turn using the same sub‐headings as Hette‐Tronquart.

## Feeding Strategy and Stable Isotopes

Hette‐Tronquart highlights that stable isotope measures integrate dietary information over the time period which the analysed tissue was synthesised (sampling period). Therefore, the multiple stable isotope values used to create our relative individual niche index (RINI) measure ‘the variability of diet over the sampling period’ and so we may not be able to differentiate between feeding strategies that vary over shorter or longer timescales than the sampling period. We recognised this in our study and acknowledge again that when interpreting the ecological meaning of stable isotope data, it is fundamental to consider this sampling period (Bearhop *et al.* 2004). In our study, ^13^C:^12^C and ^15^N:^14^N isotope ratios were measured from mongoose vibrissae that had a mean growth time of 6.3 months (lower‐upper SE = 5.3–7.8, Sheppard *et al.* 2018). Rainfall at our study site, which drives invertebrate prey abundance, fluctuates seasonally every 2–5 months (Marshall *et al.* 2017). As such, the tissues we used to calculate each RINI value indicate between‐season variation in individual diets (noting the influence of other factors below).

The timescale over which stable isotope data are measured may influence the foraging strategy they suggest. For example, one individual might always have a narrow diet but regularly switch prey items between time periods (e.g. seasons). Another individual may maintain a broader diet across these time periods that incorporates a wider range of prey items than the first individual at any given time point but not the full range of prey consumed by the first individual across all periods. Here, the first individual may appear more ‘specialist’ within a time period but more ‘generalist’ across time periods. Selection of the sampling period depends on the individual researcher judging what is ecologically relevant and what tissues are feasibly available. Future work exploring this relationship between sampling period and foraging strategy would provide valuable insights in foraging niche ecology and more broadly, the ecology and evolution of between‐individual differences in behavioural plasticity (Nussey *et al.* 2007; Dingemanse & Wolf 2013).

## Meaning of Isotopic Variability

Hette‐Tronquart suggests that the variation we observed in mongoose stable isotope ratios may be due to temporal changes in prey isotope values (‘isotopic baseline’) rather than variation in individual diet (Yeakel *et al.* 2016). However, as he points out, there is no reason to expect the isotopic baseline to vary systematically with mongoose group size (our measure of intragroup competition). This potential source of bias is, therefore, unlikely to have influenced our findings.

Hette‐Tronquart also suggest that our findings may be due to individual differences in discrimination factors since these can be affected by growth rates (Jenkins *et al.* 1999), which in turn can be influenced by competition (Gorokhova 2018). This argument is based on variation in nitrogen discrimination factors from different experimental growth rates (Gorokhova 2018) being larger than the variability in δ^15^N that Hette‐Tronquart calculates from our data. The values in Gorokhova (2018) were measured in sub‐adult marine shrimp (*Neomysis integer*) under laboratory feeding regimes (including total starvation). It is not reasonable to apply this observation to physiological processes in a wild population of, predominantly adult, mammals (mean ± SD = 3.5 ± 1.6 years, *n* = 64, Sheppard *et al.* 2018) subject to seasonal, but not extreme (e.g. starvation), changes in food availability. In addition, the δ^15^N variability measures calculated by Hette‐Tronquart include mongooses with three or fewer isotope values which were excluded from our analyses. Repeating these calculations using the 64 individuals with four or more values produces a mean ± SD δ^15^N of 1.4 ± 0.5 rather than 1.0 ± 0.6.

## Niche Overlap in Isotopic Space

Hette‐Tronquart’s final concern is that individual foraging niche sizes within a social group are not informative about the degree of niche overlap. While we expect a relationship between niche size and overlap, any ecological relationship between two variables is certain to contain a non‐trivial amount of variation caused by other factors. This is especially so when considering indirect measures of ecological processes. Stable isotope values are influenced by consumers’ diets but also by their habitat use and tissue synthesis processes. We argue that where two variables are correlated they can still provide useful information about each other as long as other sources of variation are acknowledged.

**Figure 1:**
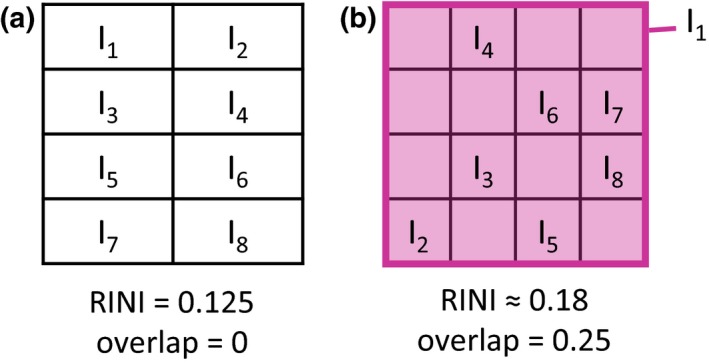
Redrawing Hette‐Tronquart’s figure 2A. Isotopic niches of individuals (I_1‐8_) within a social group showing the mean relative individual niche index (RINI) and niche overlap in each scenario. Hette‐Tronquart’s two panels in his figure 2A contain different numbers of individuals (4 vs. 8). Here, panel (a) corrects the scenario containing four individuals to contain eight, making this comparable with panel (b). Hette‐Tronquart calculates overlap as the mean proportion of each individual’s niche occupied by another group member. Here, panels (a) and (b) show how the overlap values change when this is calculated using the proportion of other group members with which each individual’s niche overlaps.

Second, the two panels in Hette‐Tronquart’s fig. 2a used to illustrate his argument are not comparable as they contain different numbers of individuals (4 vs. 8). If both include eight individuals then RINI = 0.125 and 0.18 respectively (rather than 0.25 and 0.18; see Fig. 1). We included the proportion of individuals sampled in our models to control for this sample size effect. Hette‐Tronquart also calculates overlap as the mean area that each individual’s niche overlaps with any other. This definition of overlap considers whether competition is occurring at a given point in niche space but not the intensity of this competition (i.e. the number of competitors). As such, in the second panel of Hette‐Tronquart’s fig. 2a individuals I_2‐8_ can have niches up to seven times larger without affecting the mean overlap, as long as these niches expand into space that is already occupied. This assumes that once two individuals’ niches overlap at a particular point in niche space, a third individual’s niche at this point has a negligible effect on competition. We argue that the proportion of group members that an individual is competing with (signified by individuals’ niches overlapping) would be a more ecologically relevant measure of competition. In the scenario presented by Hette‐Tronquart this more relevant measure produces mean overlap values of 0 and 0.25 rather than 0 and 0.93 (Fig. 1).

Finally, we restate here our agreement that considering the timescale over which stable isotope data are sampled is important in their interpretation, and that our study cannot test for temporal niche partitioning. However, the RINI would support assessment of niche variation over shorter timescales (e.g. days) if the measures were based on tissue samples synthesised over shorter periods.

## Conclusion

These discussions emphasise the importance of considering tissue synthesis time and ecological relevance when analysing and interpreting stable isotope data. Stable isotope data are influenced by consumers’ diets, but also the habitats they occupy and their tissue synthesis processes. Consideration of all of these sources of variation is important when interpreting stable isotope values since, rather than being directly equivalent, they provide a quantitative indicator of trophic niche.

## Authorship

HM wrote the first draft of the manuscript, all authors contributed to revisions of this draft.

## Data Accessibility Statement

There is no data associated with this technical comment response other than the data associated with the original paper (Sheppard *et al.* 2018) that is already publicly available from the Figshare Repository: https://doi.org/10.6084/m9.figshare.5863416.
